# Analysis of risk factors for intraoperative bleeding in patients with Siewert type II esophagogastric junction adenocarcinoma treated by two minimally invasive surgeries and its influence on prognosis: a retrospective study

**DOI:** 10.3389/fonc.2024.1426349

**Published:** 2024-10-02

**Authors:** Yang Lan, Jian Shen, Ruqian Liu, Kai Jiang, Mingyuan Qiu, Shuai Wang, Zhou Lin

**Affiliations:** ^1^ Department of Gastrointestinal Surgery, Affiliated Hospital of Jiangnan University, Wuxi, China; ^2^ Department of Cardiothoracic Surgery, Affiliated Hospital of Jiangnan University, Wuxi, China

**Keywords:** Siewert type II, adenocarcinoma of the esophagogastric junction, laparoscopy-assisted abdominal trans-hiatal method, transthoracic laparoscopic esophagectomy, intraoperative bleeding

## Abstract

**Background:**

The present study aimed to analyze the independent risk factors for intraoperative bleeding in Siewert II adenocarcinoma of the esophagogastric junction (AEG) using two minimally invasive surgical approaches, namely, the laparoscopy-assisted abdominal trans-hiatal (LTH) method and transthoracic-laparoscopic esophagectomy (TLE).

**Methods:**

The clinical data of 100 patients with SiewertII AEG admitted to our hospital from October 2017 to October 2020 were retrospectively analyzed. According to the type of surgery, the patients were divided into LTH approach group and TLE approach group. The differences between the clinical characteristics of the patients in different groups and the differences in the intraoperative bleeding and prognosis between different surgical procedures were analyzed and compared using the t-test and chi-squared test. Multiple linear regression was used to identify the independent risk factors affecting the amount of intraoperative bleeding in patients.

**Results:**

The results of this study showed that patients in the LTH group had significantly less intraoperative bleeding and operative time and significantly better postoperative recovery than the TLE group. The results of multivariate linear regression showed that the combined trans-thoracic-abdominal approach (P=0.000), advanced age (P=0.014), larger BMI (P=0.000), and larger tumor diameter (P=0.001) were the independent risk factors influencing the increase in intraoperative bleeding.

**Conclusion:**

In addition to the conventional factors that affect intraoperative bleeding, such as the patient’s general condition, operation time, and tumor size, LTH surgery is another way to avoid intraoperative bleeding for Siewert type II AEG patients and can significantly improve postoperative recovery.

## Introduction

Siewert II stage adenocarcinoma of the esophagogastric junction (AEG) is the most common in China, where type II denotes that the tumor center is located within 1 cm above on the Z-line to 2 cm below the Z-line, which is referred to as true cardia carcinoma (1–5). Studies conducted in China demonstrated that the proportion of AEG in all gastric adenocarcinomas increased from 22.3% in the early 1990s to 35.7% at present (6, 7). Since AEG is located at the junction of the esophagus and stomach and is a malignant tumor, it leads to lymphatic metastasis. Therefore, the effectiveness of surgical access and resection, standardized regional lymph node dissection, and GI reconstruction remains debatable.

In comparison to traditional open surgery, laparoscopic techniques offer the advantage of minimal invasion, which leads to better safety and rapid postoperative recovery (8–10). In recent years, laparoscopic techniques have been applied to Siewert II AEG as well. Thoracolaparoscopic esophagectomy (TLE) is a micro-innovative exploratory procedure, which has emerged in recent years and allows for accurately locating the lesion site and further thoroughly clearing the tumor lymph nodes, causes less damage to the nerves, and leads to less intraoperative bleeding and relatively rapid postoperative recovery (11). The other type, i.e., the laparoscopic trans-hiatal esophagectomy (LTH), is also used widely in the treatment of Siewert II AEG. Sugita et al. (12) demonstrated that laparoscopic trans-hiatal esophagectomy for Siewert II AEG has good oncologic safety. In comparison to open trans-hiatal esophagectomy (OTH), LTH has a longer operative duration, clears a significantly higher number of lymph nodes, reduces intraoperative bleeding, decreases postoperative pain, and accelerates the feeding time (12, 13).

However, a great deal of controversy remains regarding the safety and effectiveness of surgical access for Siewert type II AEG (14). In the Japanese clinical study JCOG9502, the cases of Siewert type II and III AEG were randomly divided into the left-sided combined thoracoabdominal incision group and the transabdominal esophageal fissure group, and it was revealed that the left-sided combined thoracoabdominal incision group had a significantly higher incidence of blood transfusion (15, 16). In a single-center prospective study conducted in the United Kingdom, which compared right thoracoabdominal open surgery with total laparoscopy combined with thoracoscopy. No statistically significant differences were observed in the incidence of postoperative anastomotic leak, R0 resection rate, or the number of lymph nodes dissected between the two surgical approaches used in that study, and bleeding was significantly reduced in the total laparoscopic group (300 mL *vs*. 400 mL, P = 0.021) (17).

The existing studies in the literature on minimally invasive techniques for the treatment of Siewert II AEG are mostly focused on comparison with traditional open surgery in terms of treatment outcomes. In this context, the present study aimed to identify the independent risk factors for intraoperative bleeding by analyzing and comparing the data on intraoperative indicators, perioperative indicators, and postoperative complications between the above-stated two surgical approaches. The findings of the present study would serve as a reference for the minimally invasive treatment of Siewert II AEG.

## Materials and methods

### Study participants

A total of 100 patients with Siewert II AEG who underwent minimally invasive surgery at the Affiliated Hospital of Jiangnan University between October 2017 and October 2020 were included in the present study.

The inclusion criteria were as follows: (I) postoperative pathological diagnosis of Siewert type II AEG (the tumor center located within 1 cm above on the Z-line to 2 cm below the Z-line); (II) p T1~4 N0~3M0 according to the pathological TNM staging (8^th^ edition) of the American Joint Committee on Cancer (AJCC); (III) no history of esophageal or gastric surgery (including the history of endoscopic surgery); (IV) no history of laparoscopic or combined thoracoabdominal surgery (including the history of endoscopic surgery); (V) American Society of Anesthesiologists (ASA) score ≤ 2; (VI) complete preoperative and postoperative findings, surgical records, and pathological data.

The exclusion criteria were as follows: (I) invasion of surrounding tissues and organs (T4b) or distant transfer (M1); (II) exploratory surgery, palliative resection, or combined organ resection; (III) ASA score≥3; (IV) combination of malignant tumors in other organs; (V) having received preoperative or perioperative immunotherapy, chemotherapy, radiotherapy; (VI) missing data.

**Figure 1:**
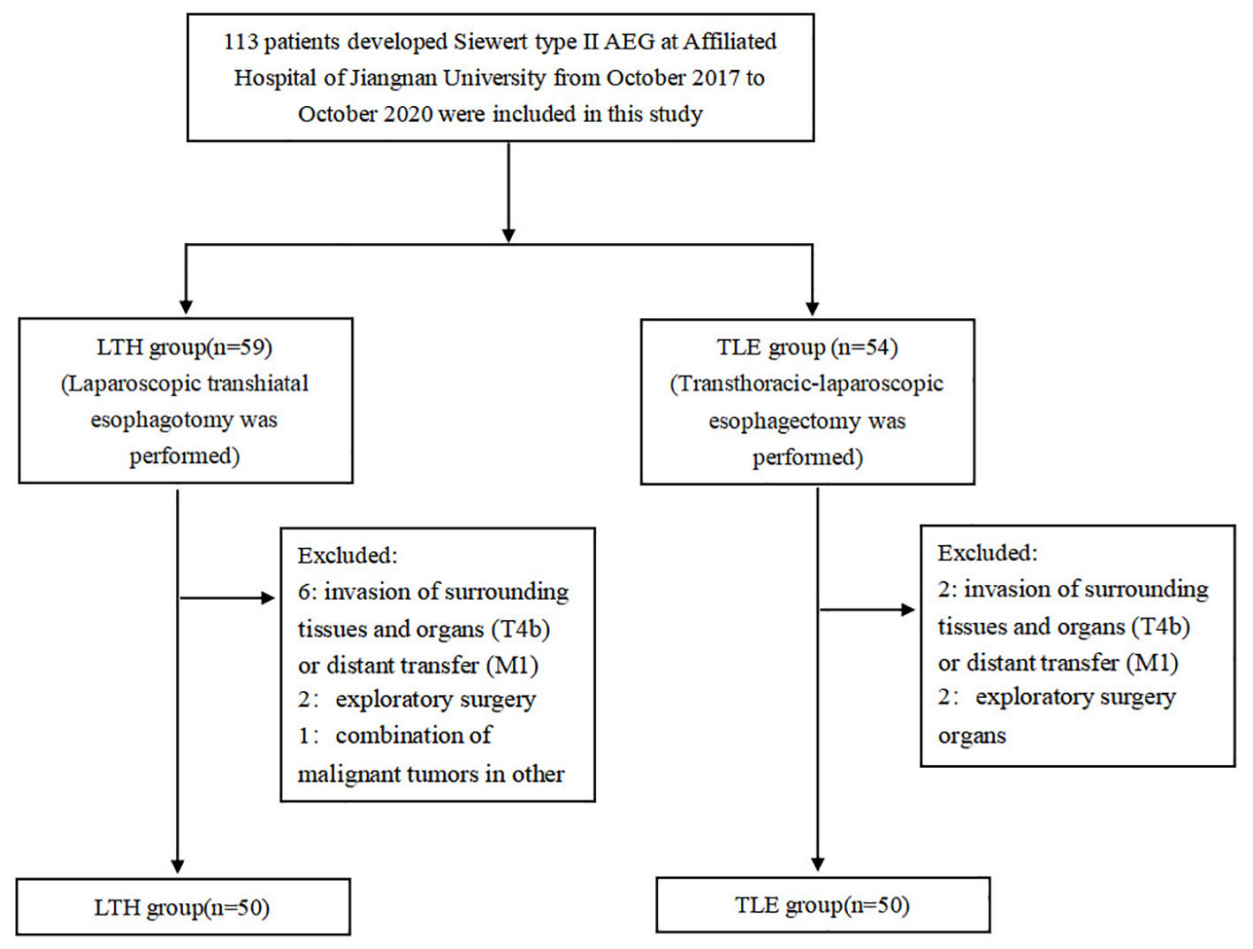
Flow chart of patient selection.

This study was a retrospective study in which patients were categorized into the laparoscopic-assisted abdominal trans-hiatal (LTH) approach group and the transthoracic-laparoscopic esophagectomy (TLE) approach group according to the type of surgical procedure they underwent. We included a total of 113 patients with Siewert II AEG who underwent surgery at our institution during the study period all together, and after screening by the inclusion and exclusion criteria, 100 patients were included in the study, with 50 patients in each group (**Figure 1**).

### General information questionnaire

The general information questionnaire included demographic data [e.g., age, gender, body mass index (BMI), smoking history, and alcohol history] and clinical data (presence of combined hypertension, diabetes, and hyperlipidemia, ASA score, tumor diameter, pathological grade, p TNM stage, presence of vascular invasion, and presence of neurological invasion) of the patients.

### Surgery method

In the LTH group, laparoscopic surgery was performed using the 5-hole approach to the upper abdominal wall under routine general anesthesia and with the establishment of a pneumoperitoneum. After the laparoscopic transabdominal exploration for resectable tumors, D2 lymph node dissection was performed (1). The diaphragmatic fissure was opened to expose the esophagus upward, and the lymph nodes around the abdominal segment of the esophagus and the lower mediastinum were cleared. A small incision was performed in the middle of the abdomen to enter the abdominal cavity, and the esophagus was dissected at a minimum distance of 2 cm from the upper edge of the tumor. The gastric body was dissected at a minimum distance of 5 cm from the lower edge of the tumor. Next, distal esophagectomy + proximal gastrectomy + esophagogastric anastomosis or distal esophagectomy + total gastrectomy + esophagojejunostomy Roux-en-Y anastomosis was performed. Afterward, the abdominal drainage tube was left in place, and the abdomen was closed layer by layer.

In the TLE group, the patient was anesthetized intravenously while the patient was lying flat, and the following five perforations were established in the abdomen: 0.5 cm and 1.5 cm operating holes at the junction of the left and right midclavicular lines, a 3 cm horizontal line above the umbilicus, 0.5 cm operating holes at the left and right assisted subxiphoid, and a 1 cm laparoscopic hole along with an incision under the umbilicus for artificial air. The lesser omentum was opened laparoscopically, the large and small curvatures of the stomach were exposed laterally to the esophageal fissure, and the lymph nodes adjacent to the left gastric vessels were cleared. The left gastric vessel was ligated, and the greater omentum was excised. The stomach was cut and sutured endoscopically to form it into a tubular stomach, and each abdominal incision was closed. In the thoracoscopic part of the surgery, three operating holes were established with the patient lying in the right lateral position: a 1.5 cm laparoscopic hole in the ninth intercostal space along the axillary line, a 4 cm operating hole in the seven assisted intercostal spaces in the mid-axillary line, and a 1.5 cm auxiliary operating hole in the intercostal space in the shoulder line. Next, the lower esophagus was exposed to the level of the pulmonary vein under the laparoscope, the lymph nodes next to the upper and lower esophagus were cleared. The esophagus was dissected at a minimum distance of 2 cm from the upper edge of the tumor, the stomach was lifted into the thoracic cavity through the diaphragmatic fissure, and the gastric body was cut at a minimum distance of 5 cm from the lower edge of the tumor. Afterward, distal esophagectomy + proximal gastrectomy + esophagogastric anastomosis + gastrojejunostomy Roux-en-Y anastomosis was performed, and the stump was closed with a cutting suture. A chest tube and mediastinal latex tube were placed, and the incision was closed after resetting the gastric tube. After surgery, upper abdominal imaging examination or review were performed routinely in the two groups to evaluate postoperative recovery and whether there were postoperative complications.

### Karnofsky performance score

The Karnofsky score (KPS) reflects the patient’s condition, ability to perform normal activities, and degree of self-care (2). The higher the score, the better the health status of the patient and the more is the patient able to tolerate the side effects of the treatment. The specific scores are as follows: 100: normal with no signs and symptoms; 90: able to perform normal activities and with just minor signs and symptoms; 80: barely able to perform normal activities and has a few signs and symptoms; 70: able to take care of themselves although cannot maintain normal life and work; 60: mostly able to take care of themselves, although require occasional assistance; 50: often require care; 40: unable to take care of themselves and require special care and help; 30: life is seriously affected, and the patient is unable to take care of self; 20: seriously ill, requiring hospitalization and active supportive treatment; 10: critically ill, near death; 0: death.

The patients were evaluated at different follow-up time points of postoperative months 1, 3, and 6, with the last follow-up in April 2021.

### Follow-up visits

All patients were routinely followed up with regular postoperative outpatient follow-up visits or telephone calls or text messages. The follow-ups were scheduled for once every 3 months each year until death or until loss of follow-up. The last follow-up visit was scheduled for December 2023.

### Statistical analysis

The results of each scale were input into the computer for score conversion. The measurement data were expressed as mean and standard deviation while numeric data were expressed as frequency and percentage. Statistical analysis of the result data was performed using SPSS 26 (IBM SPSS, USA). The differences between the groups were evaluated using the t-test and chi-squared test. After covariate diagnosis, independent risk factors for intraoperative bleeding were determined through multiple linear regression analysis. A two-sided p-value of <0.05 was considered the threshold of statistical significance.

## Results

### Baseline data

No statistical difference (P > 0.05) in age, BMI, gender, presence of combined underlying diseases, smoking history, drinking history, ASA classification, presence of *H. pylori* infection, tumor diameter, pathological classification, TNM stage, the occurrence of vascular invasion, and the occurrence of nerve invasion was observed between the two groups (**Table 1**).

**Table 1:** Baseline data of included patients with different surgery method.

Item N(%)	TLE group	LTH group	t/X^2^	P
Age (years) (Mean ± SD)	64.20 ± 8.08	62.36 ± 8.59	-1.103	0.273
BMI (kg/m^2^) (Mean ± SD)	21.29 ± 2.67	20.87 ± 3.27	-0.704	0.483
Gender				
Male	23(46.0)	26(52.0)	0.360	0.548
Female	27(54.0)	24(48.0)		
With hypertension or not				
Yes	16(32.0)	13(26.0)	0.437	0.509
No	34(68.0)	37(74.0)		
With diabetes or not				
Yes	9(18.0)	11(22.0)	0.250	0.617
No	41(82.0)	39(78.0)		
History of smoking				
Yes	16(32.0)	13(26.0)	0.437	0.509
No	34(68.0)	37(74.0)		
History of alcohol				
Yes	20(40.0)	17(34.0)	0.386	0.534
No	30(60.0)	33(66.0)		
*Hp.* infection				
Yes	33(66.0)	28(56.0)	1.051	0.305
No	17(34.0)	22(44.0)		
Tumor diameter				
≤4cm	26(52.0)	30(60.0)	0.649	0.420
>4cm	24(48.0)	20(40.0)		
Pathological grading				
Low divergence	27(54.0)	31(62.0)	0.657	0.418
Middle/high divergence	23(46.0)	19(38.0)		
p TNM staging				
I-II	33(66.0)	37(74.0)	0.762	0.383
III	17(34.0)	13(26.0)		

TLE, transthoracic-laparoscopic esophagectomy; LTH, laparoscopic-assisted abdominal-transhiatal; BMI, Body mass index; ASA, American Society of Anesthesiologists; Hp. Helicobacterpylori.

### Comparison of the intraoperative index in patients who underwent different surgical approaches

**Figure 2:**
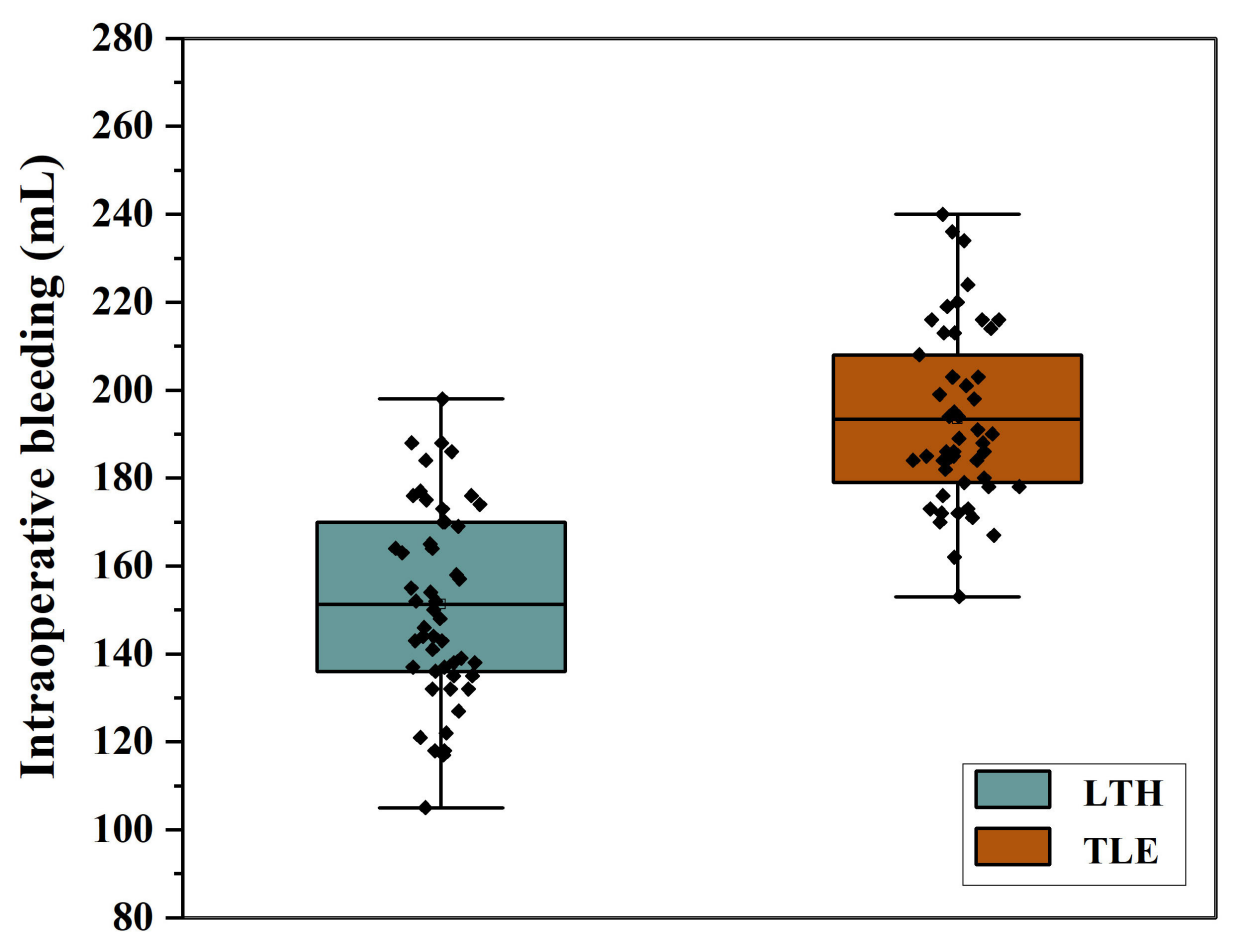
Boxplot of intraoperative blood loss in the two groups.

The t-test results revealed that the bleeding volume, operative duration, and proximal margin distance were significantly different between the transabdominal group and the combined thoracoabdominal group (P < 0.05). The LTH group had significantly less bleeding, operative time and proximal margin distance than the TLE group (**Table 2**, **Figure 2**).

**Table 2:** Comparison of intraoperative index in patients with different surgery method.

Item (Mean ± SD)	Intraoperative bleeding (mL)	Operative time (min)	Number of lymph nodes dissected	Number of positive lymph nodes	Proximal margin distance (cm)
LTH group	151.32 ± 21.87	220.74 ± 23.16	22.82 ± 6.43	4.46 ± 3.10	3.35 ± 0.70
TLE group	193.34 ± 19.89	304.92 ± 25.09	23.06 ± 6.61	4.82 ± 2.91	3.85 ± 0.87
t/z	-10.051	-17.435	-0.184	-0.599	-3.203
P	0.000	0.000	0.854	0.511	0.002

TLE, transthoracic-laparoscopic esophagectomy; LTH, laparoscopic-assisted abdominal-transhiatal.

### Comparison of the perioperative indicators in patients who underwent different surgical approaches

The t-test results revealed that the time to first bed activity, time to begin the intake of postoperative fluids, and time to postoperative hospitalization were significantly different between the LTH group and the TLE group (P < 0.05). The LTH group had a significantly shorter time to first bed activity, time to start postoperative fluids, and postoperative hospitalization than the TLE group (**Table 3**).

**Table 3:** Comparison of perioperative indicators in patients with different surgery method.

Item (Mean ± SD)	First time out of bed activity (d)	Time of drainage tube removal (d)	Time of starting liquid diet after surgery (d)	Time of postoperative hospitalization (d)
LTH group	2.58 ± 0.79	9.72 ± 0.81	4.50 ± 1.15	11.34 ± 1.59
TLE group	3.04 ± 0.73	9.72 ± 0.78	7.44 ± 1.07	13.96 ± 1.40
t	-3.040	0.000	-13.239	-8.760
P	0.003	1.000	0.000	0.000

TLE, transthoracic-laparoscopic esophagectomy; LTH, laparoscopic-assisted abdominal-transhiatal.

### Comparison of the laboratory indicators in patients who underwent different surgical approaches

The t-test results revealed that Hb, CEA, CA125, D-dimer, FDP, Alb, and PGR were not significantly different (P > 0.05) between the LTH and TLE groups (**Table 4**).

**Table 4:** Comparison of laboratory indicators in patients with different surgery method.

Item (Mean ± SD)	Hb (g/L)	CEA (ng/ml)	CA125 (ng/ml)	D-dimer (μg/ml)	FDP (μg/ml)	Alb (g/L)	PGR
LTH group	109.57 ± 18.74	26.53 ± 11.03	77.39 ± 7.14	0.80 ± 0.30	6.91 ± 0.92	40.16 ± 8.40	5.36 ± 1.24
TLE group	106.35 ± 19.16	27.36 ± 11.58	79.07 ± 6.60	0.86 ± 0.32	6.82 ± 0.88	38.26 ± 11.37	5.27 ± 1.38
t	0.848	-0.367	-1.220	-1.128	0.449	0.947	0.313
P	0.398	0.714	0.225	0.262	0.654	0.346	0.755

TLE, transthoracic-laparoscopic esophagectomy; LTH, laparoscopic-assisted abdominal-transhiatal; Hb, Hemoglobin; CEA, Carcinoembryonic antigen; CA125, Carbohydrateantigen125; FDP, Fibrinogen degradation product; PGR, Pepsinogen rate.

### Comparison of complications in patients who underwent different surgical approaches

The results of the chi-squared test revealed that only fistula formation and lung infection among the complications were significantly different between the LTH and TLE groups (P < 0.05). In the LTH group, no patients developed a pulmonary infection, while 7 (14.0%) developed postoperative fistula formation. In the TLE group, 4 (8.0%) developed a pulmonary infection, and just 1 (2.0%) developed postoperative fistula formation (**Table 5**).

**Table 5:** Comparison of complications in patients with different surgery method.

Item N(%)	Abdominal infection		Pulmonary infection		Fistula formation		Anastomotic bleeding		Intestinal obstruction		Incisional infection	
	Yes	No	Yes	No	Yes	No	Yes	No	Yes	No	Yes	No
TLE group	0(0.0)	50(100.0)	4(8.0)	46(92.0)	1(2.0)	49(98.0)	2(4.0)	48(96.0)	1(2.0)	49(98.0)	1(2.0)	49(98.0)
LTH group	2(4.0)	48(96.0)	0(0.0)	50(100.0)	7(14.0)	43(86.0)	1(2.0)	49(98.0)	1(2.0)	49(98.0)	0(0.0)	50(100.0)
X^2^	2.041		4.167		4.891		0.344		0.000		1.010	
P	0.153		0.041		0.027		0.558		1.000		0.315	

TLE, transthoracic-laparoscopic esophagectomy; LTH, laparoscopic-assisted abdominal-transhiatal.

### Risk factors of intraoperative bleeding analyzed using multiple linear regression models

The adjusted R-squared value was 0.822, the Durbin-Watson coefficient was 1.872, and the maximum value of VIF (variance inflation factor) was 1.434. The residuals were distributed normally. No significant autocorrelation was observed between the variables after covariance diagnosis. The results of multiple linear regression showed that the TLE approach, advanced age, greater BMI, comorbid hypertension, comorbid diabetes mellitus, comorbid Hp infection, and greater tumor diameter were independent risk factors affecting the increase of intraoperative bleeding (**Table 6**).

**Table 6:** Risk factors of intraoperative bleeding analyzed by multiple linear regression models.

Item	B	SE	t	P	VIF
Surgery method	37.774	2.595	14.555	0.000	1.074
Age (years)	0.450	0.180	2.496	0.014	1.434
BMI (kg/m^2^)	1.778	0.450	3.947	0.000	1.135
Gender	0.904	2.871	0.315	0.754	1.314
With hypertension or not	9.264	2.925	3.167	0.002	1.124
With diabetes or not	13.564	3.627	3.739	0.000	1.343
History of smoking	1.641	2.951	0.556	0.580	1.143
History of alcohol	3.319	2.806	1.183	0.240	1.171
*Hp.* infection	13.202	2.966	4.451	0.000	1.335
Tumor diameter	10.326	3.011	3.430	0.001	1.425
Pathological grading	-3.445	2.949	-1.168	0.246	1.351
p TNM staging	-0.094	2.872	-0.033	0.974	1.105

BMI, Body mass index; ASA, American Society of Anesthesiologists; Hp. Helicobacterpylori.; Hb, Hemoglobin; CEA, Carcinoembryonic antigen; CA125, Carbohydrateantigen125; FDP, Fibrinogen degradation product; PGR, Pepsinogen rate; SE, standard error; VIF, variance inflation factor.

### Karnofsky score of the patients in the two groups during follow-up

The t-test results revealed a significant difference between the LTH group and the TLE group in the KPS scores assessed at one month postoperatively (P < 0.05), while no significant difference was observed between the two groups in the KPS scores assessed preoperatively, 3 months postoperatively, and 6 months postoperatively (P > 0.05). The LTH group had a significantly higher KPS score at one month postoperatively than the TLE group (**Table 7**).

**Table 7:** Karnofsky score of patients in both groups of follow-up.

Item (Mean ± SD)	Before surgery	1 month after surgery	3 month after surgery	6 month after surgery
LTH group	59.92 ± 7.73	73.76 ± 8.81	80.42 ± 8.07	83.32 ± 6.897
TLE group	58.76 ± 7.56	70.32 ± 7.55	77.74 ± 7.41	81.74 ± 6.75
t	0.750	2.097	1.729	1.158
P	0.450	0.039	0.087	0.250

TLE, transthoracic-laparoscopic esophagectomy; LTH, laparoscopic-assisted abdominal-transhiatal.

## Discussion

The patients with Siewert type II AEG are currently treated using two main clinical surgical routes –thoracic surgery, with the TLE approach usually adopted and performed according to the guidelines for the treatment of esophageal cancer, and the general surgery, with the LTH approach usually adopted and performed with reference to the guidelines for the treatment of gastric cancer (4). However, no consensus has been reached so far on the best choice among the two approaches (8). Irrespective of which surgical approach is selected, intraoperative bleeding is inevitable, also has a significant effect on prognosis (9, 10).

The results of the multiple linear regression analysis conducted in the present study revealed the TLE approach, advanced age, greater BMI, comorbid hypertension, comorbid diabetes mellitus, comorbid Hp infection, and greater tumor diameter as the independent risk factors for increased intraoperative bleeding. Xing et al. (4) reported that the laparoscopy-assisted transabdominal diaphragmatic fissure approach leads to shorter operative duration, less intraoperative bleeding, and rapid postoperative recovery compared to the combined thoracoabdominal approach, which is consistent with the results of the present study. Since elderly patients have several preoperative comorbidities and poor surgical tolerance, the incidence of perioperative complications in these patients is higher (11). In particular, a high incidence of cardiovascular and metabolic diseases leads to endovascular sclerosis and increased vascular fragility, which causes intraoperative dissection to bleed easily. Obesity remains a worldwide problem at present (12). The large amount of fat in the abdominal cavity of obese patients and the brittle texture of this fat renders bleeding more probable during clamping or pulling, which affects the surgical operation and vision. In addition, the large amount of fatty tissue wrapping the blood vessels in the surgical region interferes with the surgeon’s accurate prediction of the location of the blood vessels in that surgical region which again renders it easy to have intraoperative bleeding. It is reported that obesity is an independent risk factor for intraoperative bleeding in laparoscopic gastric cancer (13). In their report, Yoshikawa et al. (14) stated that in 66 cases of laparoscopic distal gastric cancer radical treatment, the mean intraoperative bleeding was 148 mL in obese patients and 48 mL in non-obese patients, and the values were statistically different. Li et al. (15) conducted a meta-analysis of 18,518 cases of gastric cancer surgeries and reported that BMI > 25 was the most important independent risk factor for intraoperative bleeding in gastric cancer. HP infection colonizes the gastric mucosa of patients, releasing huge amounts of metabolic toxicants, such as esterase and pepsin, which damage the protective barrier of the gastric mucosa (16). It also induces the release of various inflammatory factors, leading to an inflammatory response, which aggravates gastric mucosal damage, ultimately increasing the incidence of hemorrhage (17, 18). In addition, HP adherent epithelial cells could reduce the microvilli, causing the disappearance of intercellular connections, depleting the mucus granules in the cells, and exhibiting vacuole-like changes, thereby leading to the formation of shallow cup-like, adherent tip structures between the cells and the bacteria, which increases the incidence of peptic ulcers that are ultimately predisposed to intraoperative bleeding (19, 20). In addition, larger lesions are a common risk factor for intraoperative bleeding. Certain studies have demonstrated that the probability of intraoperative bleeding is greatly increased in lesions larger than 4 cm in diameter (21). This could be attributed to the fact that lesion with larger diameters increase the extent of surgical resection and trauma to the body, which could increase the intraoperative bleeding. Moreover, the esophagogastric junction is rich in submucosal vascular structures, lacks a plasma membrane layer, has a weak muscular layer, and is also affected by the physiological peristalsis of the lumen, anatomical structures, heartbeat, and respiration. These limits the operating space and field of vision during the surgery, thereby increasing the difficulty of the surgical operation and increasing the risk of intraoperative vascular and tissue injury, ultimately predisposing the region to intraoperative bleeding. Operative duration is an important factor influencing intraoperative bleeding, although few studies in the literature confirm this view. KukrejaR et al. conducted a study on percutaneous nephrological surgery and concluded that operative duration was an independent risk factor for postoperative hemoglobin reduction (22). Akman T et al. also concluded that operative duration was an independent risk factor for intraoperative transfusion required in percutaneous nephrological surgery (23). This could be attributed to the relatively great trauma suffered by the body during the extended operative duration, which promoted the release of a large number of inflammatory factors, thereby leading to the inhibition of the coagulation factors. In addition, with the prolonged operative duration, the level of inflammatory mediators in the body increases, thereby promoting vasodilation and ultimately increasing the risk of intraoperative bleeding (24, 25). Prolonged exposure to surgery may induce a stress response in the body, which may cause increased vascular permeability and a significant increase in the levels of oxygen free radicals generated, which could largely increase the incidence of intraoperative bleeding (26, 27). The present study also investigated the effects of different surgical approaches on patient prognosis. First, the results revealed that the time to first bed activity etc, were longer in the combined thoracoabdominal group than in the transabdominal group. This finding indicated that postoperative recovery was generally faster in the transabdominal group. Mine et al. (28) studied the effect of proximal margin length on prognosis and concluded that a proximal margin length of >2.0 cm was an independent prognostic factor. In contrast, the limited length of the esophagus resected using the transdiaphragmatic fissure approach compared to that observed using the combined thoracoabdominal group suggested that the combined thoracoabdominal approach was superior to the transdiaphragmatic fissure approach in ensuring the oncologic safety of the proximal cut edge (29).

As with all research, the present study also had certain limitations. For instance, the present study was designed as a single-center retrospective study with more confounding factors, and the data on the length of esophageal invasion and the proximal cut margin distance were obtained from the description of the sent pathological specimens. Follow-up studies can further improve the relationship between incisal margin distance and prognosis. In addition, we did not take into account the effect of Circumferential radial margin status on the results of the study. Finally, a longer follow-up period is needed to evaluate the postoperative survival of patients in both groups.

## Conclusion

In addition to the conventional factors that affect intraoperative bleeding, such as the patient’s general condition, operation time, and tumor size, LTH surgery is another way to avoid intraoperative bleeding for Siewert type II AEG patients and can significantly improve postoperative recovery.

## Data Availability

The original contributions presented in the study are included in the article/**Supplementary Material**. Further inquiries can be directed to the corresponding author.
